# The high hourly overnight variability of insulin requirements as an explanation for the superiority of automated insulin delivery systems

**DOI:** 10.1111/dom.16319

**Published:** 2025-03-07

**Authors:** Giona Castagna, Giuseppe Lepore, Nicolò Diego Borella, Roberto Trevisan

**Affiliations:** ^1^ Unit of Endocrine Diseases and Diabetology, Department of Medicine ASST Papa Giovanni XXIII Bergamo Italy; ^2^ Department of Medicine Università degli Studi di Milano‐Bicocca Milan Italy

**Keywords:** insulin pump therapy, insulin therapy, real‐world evidence, type 1 diabetes

## 1 BACKGROUNDS

Maintaining glycaemic status as close to the normal range as safely possible is the primary goal in managing type 1 diabetes (T1D) to prevent both short‐and long‐term complications.^1^, ^2^ Despite significant advances in diabetes technologies and therapeutics, a substantial proportion of individuals with T1D fail to achieve the glycaemic targets recommended by international guidelines.^3^ Automated insulin delivery (AID) systems, integrating continuous glucose monitoring (CGM) with algorithm‐driven insulin pumps, represent the most advanced approach to insulin delivery currently available. Several studies have demonstrated the effectiveness of AID systems in achieving glycaemic targets in the real world.^4^, ^5^, ^6^ We have recently demonstrated in a comparative study the effectiveness of three AID systems in achieving glycaemic metrics targets during the night‐time.^7^ However, little is known about the variability of insulin requirements, especially during the night, and investigating this issue could be important for developing improvements in AID algorithms and performance.

To our knowledge, only two studies have evaluated the variability of insulin requirements in adults with T1D using closed‐loop systems.^8^, ^9^ Our retrospective study aimed to evaluate the overnight hourly variability in insulin requirements (00:00/07:00 AM) across different nights in adults with T1D using AID systems.

## 2 METHODS

We analysed data from 55 adults with T1D followed at the Unit of Endocrine Diseases and Diabetology, ASST Papa Giovanni XXIII Bergamo, Italy. Participants (males 22 [40%]; mean age 41.2 ± 15.6 years; BMI 24.1 ± 4.1 kg/m^2^; duration of diabetes 19.4 ± 11.4 years; daily insulin requirements 0.6 ± 0.3 U/kg) were required to have used an AID system for at least 6 months prior to the study. Twenty‐two participants used the MiniMed 780G system, 18 used the Tandem t:slim X2 with Control‐IQ and 15 used the DBLG1 system. Inclusion criteria required adequate daily glycaemic control with a Glucose Management Index (GMI) between 7.0% and 7.4% over the 3 months prior to night‐time data collection.

Additionally, during the selected 14‐day study period, the absence of recorded manual boluses suggested that participants had completed dinner before 9 PM and delayed breakfast until after 7 AM.

Continuous glucose monitoring derived metrics of 14 consecutive nights, including time in range 70–180 mg/dL (TIR), time below range < 70 mg/dL (TBR) and glycaemic coefficient of variation (CV), were evaluated for each participant for a total of 770 nights. The hourly insulin requirements of participants were recorded nightly over the 14‐day study period by downloading data from insulin pumps. The coefficient of variation (CV) of nightly hourly insulin requirements was then calculated for each participant to assess inter‐night variability.

## 3 RESULTS

All participants achieved the recommended night‐time glycaemic metrics: mean TIR was 73.4 ± 11.1%, TBR 1.0 ± 2.6% and glycaemic CV 31.7 ± 7.1%. A progressive reduction in mean glucose values was observed from 179.9 ± 22.7 mg/dL at midnight to 138.0 ± 19.0 mg/dL at 7:00 AM, with stable and low glycaemic CVs during the entire night (29.1 ± 9.0% at midnight to 25.2 ± 9.4% at 7:00 AM).

**FIGURE 1:**
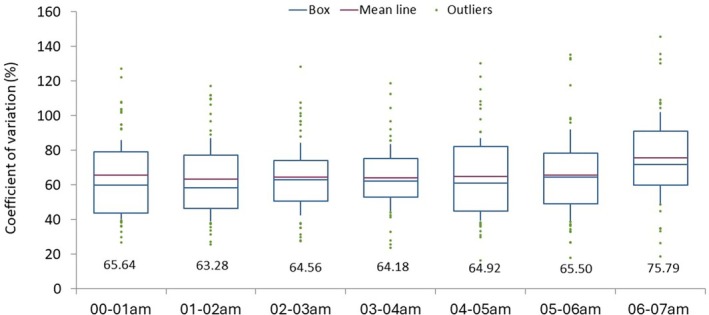
Box plots of coefficient of variation (CV) of hourly insulin requirements in 55 adults with type 1 diabetes mellitus showing the mean, median, outliers, interquartile range, and the 5th and 95th percentiles.

Overnight insulin requirements averaged 0.13 ± 0.08 U/kg, ranging from 0.04 to 0.28 U/kg. The individual CV of hourly insulin requirements was consistently high, with values exceeding 63% across all participants, as illustrated in Figure 1. No significant differences in the CV of hourly insulin requirements were observed across the different night‐time hours.

Multivariate analysis explored the relationship between the CV of hourly insulin requirements and independent variables, including demographic and clinical characteristics and glycaemic metrics as described in Table 1. Glycaemic CV was the only independent variable directly correlated with the CV of hourly insulin requirements. No correlation was found between gender and the CV of hourly insulin requirements.

**TABLE 1:** Multivariate linear regression to assess the effect of demographic and glycaemic data on mean hour insulin requirement coefficient of variation (CV).

Mean hour insulin CV (%)	95% CI	*p*
Age (years)	−0.39 to 1.27	0.31
BMI (kg/m^2^)	−0.27 to 1.36	0.18
Gender (female)	−7.20 to 6.11	0.86
Duration of diabetes (years)	−0.52 to 0.24	0.47
HbA1c (mmol/mol)	−0.45 to 0.24	0.53
TIR %	−29.04 to 28.76	0.99
TAR %	−29.92 to 28.05	0.95
TBR %	−26.93 to 30.13	0.91
Glucose CV (%)	0.58 to 1.75	**<0.001**
Mean glucose (mg/dL)	−0.18 to 1.16	0.15

Abbreviations: BMI, body mass index; CI, confidence interval; HbA1c, glycated haemoglobin; TAR, time above range >250 mg/dL; TBR, time below range <70 mg/dL; TIR, time in range 70–180 mg/dL.

## 4 CONCLUSIONS

Our findings highlight an impressively high variability in hourly insulin requirements during the night in adults with T1D using AID systems. This variability was observed in a cohort with relatively good glycaemic control (GMI 53–58 mmol/mol or 7.0%–7.4%) and in conditions with minimal confounding factors such as food intake and physical activity. These results align with a previous study by Ruan et al., which demonstrated greater variability in overnight insulin requirements compared with daytime and total daily insulin needs in individuals using closed‐loop systems.^8^

The observed high variability underscores the importance of real‐time insulin delivery adjustments provided by AID systems. Traditional long‐acting basal insulins or fixed basal rates in standard insulin pumps cannot adequately address such dynamic changes, often resulting in suboptimal glycaemic control or increased risk of hypoglycaemia.

### 4.1 Limitations

The relatively small sample size may limit the generalizability of the findings. Given the opportunity to analyse only a limited sample of patients, we carefully chose a cohort that we believed to be representative of individuals with type 1 diabetes undergoing AHCL therapy. However, future studies with larger sample sizes could benefit from broadening the inclusion criteria to encompass individuals with varying degrees of glycaemic control and assessing potential correlations between night‐time insulin requirements, CV and overall glycaemic control.

Additionally, while participants were instructed to standardise meal timing and avoid manual boluses during the study period, variations in daytime physical activity and wake‐up times were not controlled. Despite these limitations, the study provides valuable insights into real‐world conditions experienced by individuals with T1D using AID systems.

## FUNDING INFORMATION

This work was partially funded by ANTHEM ‐ AdvaNced Technologies for Human‐centrEd Medicine an Initiative funded by the Ministry of Universities and Research under the “National Plan for Investments complementary to the PNRR (PNC ‐ National Complementary Plan), intervention “Research initiatives for technologies and innovative paths in health and care” ‐ DL 6 May 2021, n. 59, converted with modifications from Law 1 July 2021, n. 101, marked by the identification code PNC0000003 (ANTHEM ‐ PNC0000003 ‐ CUP: B53C22006670001) and by the Italian Ministero dell’Università e della Ricerca (MUR) PRIN 2022 PRIN 2022KZ4KMY CUP: H53D23006510006. This work reflects only the authors’ views and opinions, neither the Ministry for University and Research nor the European Commission can be considered responsible for them.

## CONFLICT OF INTEREST STATEMENT

The authors declare no conflicts of interest.

### PEER REVIEW

The peer review history for this article is available at https://www.webofscience.com/api/gateway/wos/peer‐review/10.1111/dom.16319.

## Data Availability

The data that support the findings of this study are available from the corresponding author upon reasonable request.
